# pyM^2^aia: Python interface for mass spectrometry imaging with focus on deep learning

**DOI:** 10.1093/bioinformatics/btae133

**Published:** 2024-03-05

**Authors:** Jonas Cordes, Thomas Enzlein, Carsten Hopf, Ivo Wolf

**Affiliations:** Faculty of Computer Science, Mannheim University of Applied Sciences, Mannheim 68163, Germany; Medical Faculty Mannheim, Heidelberg University, Mannheim 68167, Germany; Center for Mass Spectrometry and Optical Spectroscopy, Mannheim University of Applied Sciences, Mannheim 68163, Germany; Medical Faculty Mannheim, Heidelberg University, Mannheim 68167, Germany; Center for Mass Spectrometry and Optical Spectroscopy, Mannheim University of Applied Sciences, Mannheim 68163, Germany; Medical Faculty, Heidelberg University, Heidelberg 69120, Germany; Faculty of Computer Science, Mannheim University of Applied Sciences, Mannheim 68163, Germany

## Abstract

**Summary:**

Python is the most commonly used language for deep learning (DL). Existing Python packages for mass spectrometry imaging (MSI) data are not optimized for DL tasks. We, therefore, introduce pyM^2^aia, a Python package for MSI data analysis with a focus on memory-efficient handling, processing and convenient data-access for DL applications. pyM^2^aia provides interfaces to its parent application M^2^aia, which offers interactive capabilities for exploring and annotating MSI data in imzML format. pyM^2^aia utilizes the image input and output routines, data formats, and processing functions of M^2^aia, ensures data interchangeability, and enables the writing of readable and easy-to-maintain DL pipelines by providing batch generators for typical MSI data access strategies. We showcase the package in several examples, including imzML metadata parsing, signal processing, ion-image generation, and, in particular, DL model training and inference for spectrum-wise approaches, ion-image-based approaches, and approaches that use spectral and spatial information simultaneously.

**Availability and implementation:**

Python package, code and examples are available at (https://m2aia.github.io/m2aia)

## 1 Introduction

Feeding large amounts of data to deep neural networks during training can be as important as it can be a bottleneck in the training process. This is particularly the case in mass spectrometry imaging (MSI), a technique for label-free imaging of the spatial distribution of hundreds of molecules in tissue sections. MSI datasets are large (up to tens of gigabytes) containing high-dimensional spectral information (thousands of *m/z* bins) per pixel.

**Figure 1.**
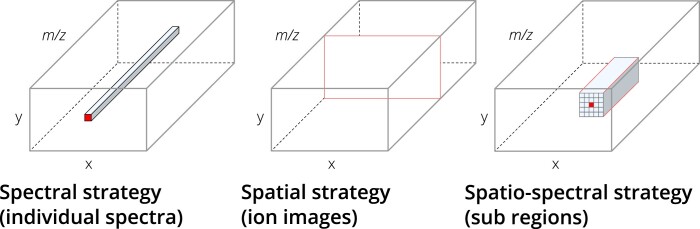
Strategies for processing MSI datasets in DL tasks. Cubes represent hyperspectral data with sample selection parameters (red) *x* and *y* for the spatial dimensions and *m/z* for the spectral dimension.

Different strategies for handling MSI data (Fig. 1) can be distinguished: (i) spectral strategy, in which the spectral information is used but the spatial relationships between spectra are lost (e.g. spectrum-wise peak picking or classification), (ii) spatial strategy, in which spatial properties of a molecular distribution are addressed but intra-spectral relationships are not taken into account (e.g. ion-image-based clustering or segmentation), and (iii) spatio-spectral strategies, which uses spatial and spectral information simultaneously, which are computationally highly demanding and still rare. For each strategy, references to example applications can be found in the Supplementary Appendix S3.

The comparatively slow progress of deep learning (DL) for MSI data may be attributed to the lack of benchmark datasets Alexandrov (2020), inconsistent data quality as a consequences of batch effects Balluff *et al.* (2021), a diverse landscape of MSI data signal processing strategies, issues related to the curse of dimensionality, and the lack of explainability and interpretability of DL models (black box). Additionally, most DL code is written in Python, whereas most MSI data science packages are written in R.

We hypothesize that the development of DL applications can best be supported by a tandem approach where interactive processing and visualization of MSI data is applied in combination with scripting for DL. Here, we introduce pyM^2^aia (biotools: pym2aia), a Python package for accessing MSI data with a focus on supporting the development of DL applications, complementing the interactive application M^2^aia (Cordes et al. 2021, biotools: m2aia).

## 2 Features

To support the development of DL applications for high-dimensional data from MSI acquisitions, pyM^2^aia aims to provide (i) memory- and computationally efficient loading of imzML datasets to make spectra or ion-images available as quickly as possible for (GPU-based) training and inference of DL models, and to facilitate the creation of (ii) readable and easy-to-maintain DL pipelines and (iii) solutions that rely on a common code-base for interactive exploration and scripting to enable consistent data views and processing.

pyM^2^aia complements the open-source desktop application M^2^aia cordes et al. (2021), which offers interactive visualization and image processing utilities (see Supplementary Table S1) for continuous profile/centroid imzML datasets. pyM^2^aia wraps the highly optimized input and processing methods (implemented in C++) of M^2^aia, enabling consistent views of MSI data in both systems. M^2^aia’s signal processing methods are directly applied after reading spectra from disk, omitting the need to hold any intermediate data in memory, which substantially increases the number of datasets that can be accessed simultaneously. The application programming interface (API) of pyM^2^aia supports high-level data handling for the implementation of DL pipelines realizing the different processing strategies defined in the introduction (Fig. 1). pyM^2^aia provides data generators for the generation of batches, enables the invocation of arbitrary data augmentation functions and allows cyclic passes over single as well as multiple MSI datasets. The API is compatible with common DL libraries like TensorFlow/Keras and PyTorch.

## 3 Results

Exemplary applications with increasing complexity were realized to showcase the capabilities of pyM^2^aia. Openly available MSI datasets are used, published by Geier *et al.* (2021). Code/data availability statements and further details on the examples can be found in the Supplementary material.

The first examples show how imzML data is handled with pyM^2^aia. In example I we demonstrate how pyM^2^aia can be used to retrieve imzML meta data. Example II illustrates the application of pyM^2^aia’s signal processing methods (Supplementary Fig. S1). Example III explains the generation of ion-images and how to overlay multiple ion-images to show co-localization of ions (Supplementary Fig. S2).

The next examples demonstrate DL applications using pyM^2^aia. Example IV showcases a *spectral strategy* that uses pyM^2^aia to feed spectra to adapted versions of an autoencoder model for peak learning proposed by Abdelmoula *et al.* (2021). The original approach is available as a TensorFlow implementation that loads datasets in *hl5* format. We replaced the *hl5* input by pyM^2^aia’s imzML reader. For stabilizing the training on the example dataset by Geier *et al.* (2021), we replaced the originally used categorical cross-entropy loss by a mean-squared error loss and removed the sigmoid activation function of the output layer. The model accepts as input a tensor of the form [B, C, H(=1),W(=1)] [with B: batch size, C: channel size (=spectral depth), H/W: height/width of patch]. The core demonstration of this example is the usage of pyM^2^aia’s spectrum batch generators (Supplementary Fig. S3). We show how to train multiple models for each imzML image individually (similar to the original publication) and how pyM^2^aia enables easily to process multiple images simultaneously (combined) to create a single model for all inputs.

A *spatial strategy* is demonstrated in example V by adapting the PyTorch implementation of an ion-image clustering approach proposed by Hu *et al.* (2022). The approach uses a pretrained EfficientNet model to embed ion-images in a latent space. The model is then fine-tuned using contrastive learning (SimCLR). SimCLR heavily relies on data augmentations, which are defined by adding augmentation functions to pyM^2^aia’s ion-image batch generator. For processing the dataset, we reduced the number of input-channels of the EfficientNet from three (RGB) to one (gray-scale) and adapt the augmentation methods to accept single-channel inputs of the form [B, C(=1),H, W]. The core demonstration of this example is to train the approach described above with pyM^2^aia’s ion-image batch generator and show how augmentations are incorporated (Supplementary Fig. S4).

*Spatio-spectral strategies* are demonstrated in example VI and example VII, showing how pyM^2^aia’s spectrum batch generator is used to generate spatio-spectral samples by providing additional neighboring spectra for a given sample location (Supplementary Fig. S5). The spectrum batch generator can retrieve specified spatial neighborhoods of size [H, W] and provides them as tensors of the form [B, C, H, W]. In example VI, we train and apply an unsupervised auto-encoder and in example VII a supervised model for pixel-wise classification.

## 4 Conclusion

pyM^2^aia gives the MSI and DL communities Python-based access to M^2^aia’s efficient implementations for MSI data handling and processing. By providing high-level convenience methods, DL workflows can be realized with less code, improving readability and reducing the risk of potential mistakes.

## Supplementary Material

btae133_Supplementary_Data
