# Supplementary material for: pyM2aia: Python interface for mass spectrometry imaging with focus on deep learning
Source: Bioinformatics. 2024 Mar 5;40(3):btae133. doi: 10.1093/bioinformatics/btae133 (PMC10948279; doi:10.1093/bioinformatics/btae133)
Supplement: btae133_Supplementary_Data [file btae133_supplementary_data.pdf]

pyM²aia: Python interface for mass spectrometry imaging data with focus on Deep Learning
Supplementary Appendix

1. MALDI-TOF-MSI DATASET

We use a data set published by Geier *et al.* (2021). Data show four sections (section 1-4) of an adult earthworm (L. rubellus) and are used in all examples. Image details of the original publication is summarized as follows: MALDI-MSI utilized a 7 mg·mL^{-1} α-cyano-4hydroxycinnamic acid matrix in 70:30 acetonitrile/water with 0.2% trifluoroacetic acid, applied by SunCollect's automated spray-coating system. Parameters included a capillary z-distance of 25 mm, compressed air pressure of 2 bar, and varying flow rates (15 μL·min^{-1} for the first layer, 20 μL·min^{-1} for layers 2-8). Imaging used an Autoflex speed LRF MALDI-TOF with smartbeam-II 1 kHz laser, a 25 μm spot size, and a "random walk" pattern. Acquisition involved 500 shots per sampling point (100 shots per location within the spot), with a mass detection range of m/z 100 to 1,280 and 200-ppm accuracy.

2. AVAILABILITY OF SUPPORTING SOURCE CODE, DATA, AND REQUIREMENTS

Data is available for download in the MetaboLights (Haug *et al.*, 2019) repository using the accession number MTBLS2639 (https://www.ebi.ac.uk/metabolights/MTBLS2639).

- Project home page: https://m2aia.github.io/m2aia
- Operating systems: Windows and Unix
- Programming language: Python version 3.8 or above
- License: BSD
- Zenodo: https://zenodo.org/doi/10.5281/zenodo.7825824
- Biotools (biotools:pym2aia): https://bio.tools/pym2aia
- [1] pyM²aia - GitHub repository: https://github.com/m2aia/pym2aia
- [2] pyM²aia-examples - GitHub repository: https://github.com/m2aia/pym2aia-examples
- [3] msiPL - GitHub fork: https://github.com/m2aia/pym2aia-examples-msiPL
- [4] MSI-self-supervised-clustering - GitHub fork: https://github.com/m2aia/pym2aia-examples-MSI-self-supervised-clustering
- Other requirements: The project is based on libraries of M²aia. Details on how to setup an working environment can be found on the GitHub project page [1].

3. RELATED WORK

- *Spectral Strategy* approaches using DL published so far include unsupervised approaches for dimensionality reduction (Thomas *et al.*, 2016) and peak picking (Abdelmoula *et al.*, 2021), as well as supervised approaches for classification (Behrmann *et al.*, 2018; Abdelmoula *et al.*, 2022).
- *Spatial Strategy* approaches using DL include clustering/co-localization of ion-images (Ovchinnikova *et al.*, 2020; Zhang *et al.*, 2021; Hu *et al.*, 2022).
- *Spatio-spectral Strategies* are still rare, with only a few applications integrating spatial and spectral information in some way, e.g. Palmer *et al.* (2017); Abu Sammour *et al.* (2021).

4. EXAMPLES

The examples provided demonstrate the practical application of pyM²aia's API and focus on the generation of MSI data samples for deep neural networks based on the strategies described in the main paper. It is important to note that these examples do not explain or evaluate the methods themselves. Detailed discussions of the methods can be found in the original publications. The first three examples show how imzML data is handled with pyM²aia. Example IV to VII demonstrate how to utilize pyM²aia for state-of-the-art DL applications. All examples can be found in the GitHub repository [2].

Example I pyM²aia enables access to image meta data, including pixel spacing (spot size), image dimensions, spectrum depth and spectrum type (continuous/processed profile/centroid spectrum types as defined in the imzML standard (Schramm *et al.*, 2012)). In addition, image context metadata, including tags and values defined in imzML, are accessible, providing detailed information about the MSI datasets.

Example II pyM²aia wraps M²aia's optimized signal processing utilities. Example II outlines how to configure the signal processing pipeline. Results of several signal processing configurations are shown in Figure S1. Currently, different types of baseline-correction, signal smoothing, normalization, pooling and intensity transformations are supported (Cordes *et al.*, 2021).

Example III Ion-image generation and how to combine multiple ion-images into a colored image (Fig. S2) is demonstrated in example III. Generated images can be written in common image formats (e.g., Nearly Raw Raster Data [*.nrrd] image file format) that are compatible with M²aia, enabling interactive exploration of image artifacts generated with pyM²aia using the desktop application M²aia.

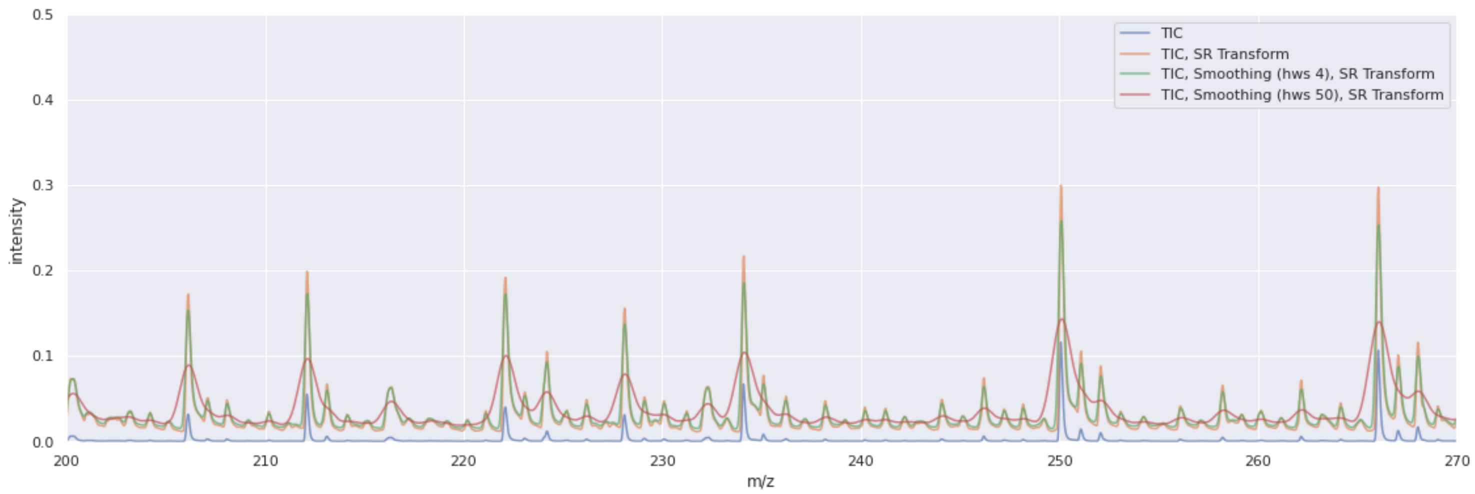

Fig. S1. Example II: comparison of mean overview spectra from the same dataset using different signal-processing methods. Mean overview spectra for Section 1 within the range of *m/z* 200 to *m/z* 270 are shown. TIC: Total Ion Current; SR: Square Root Transformation; hws: half window size.

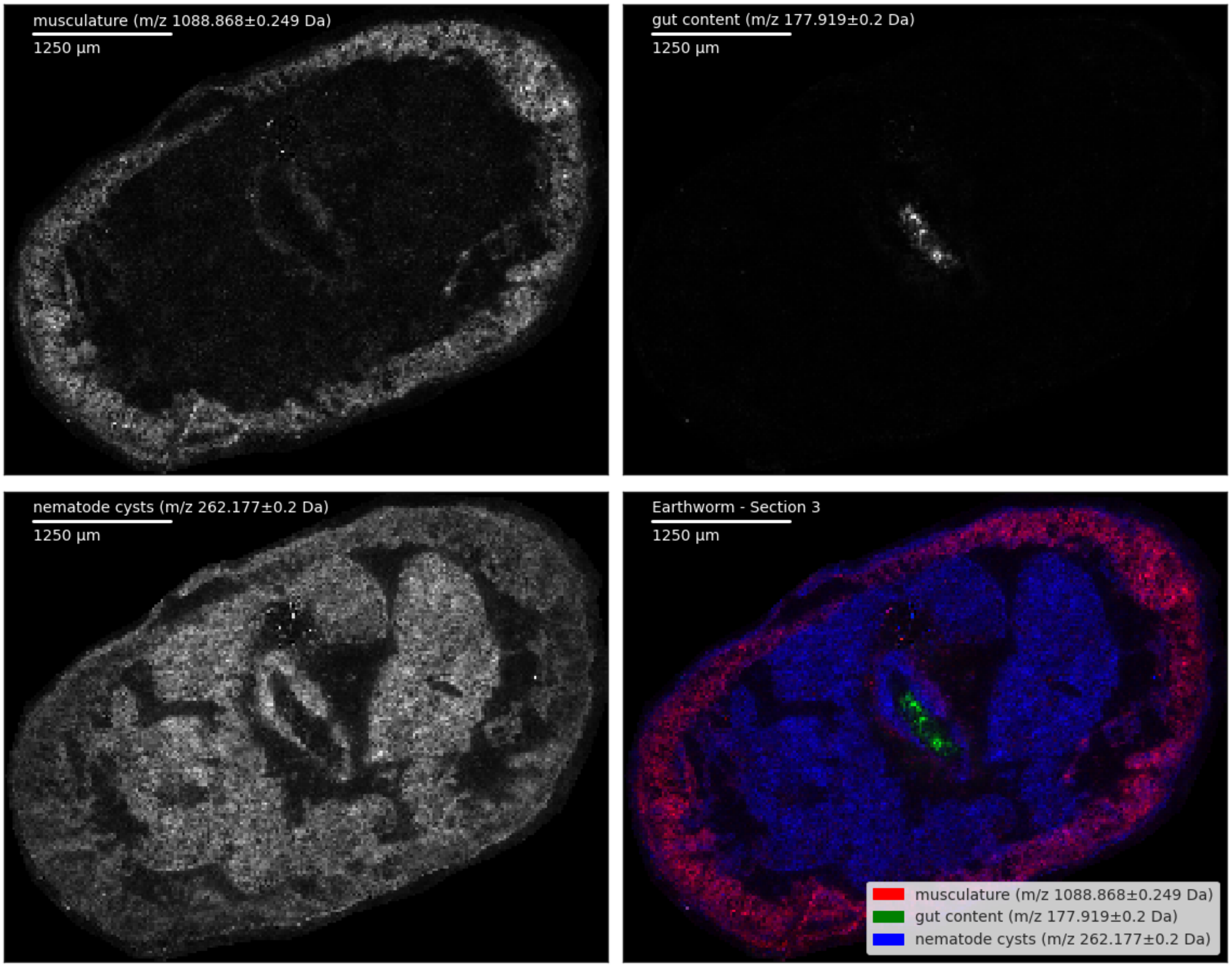

Fig. S2. Example III: earthworm sections 1-4 - combining ion-images for metabolites located within the musculature m/z 1088.868, gut content m/z 177.919, and nematode cysts m/z 262.177 to a single multi-colored representation.

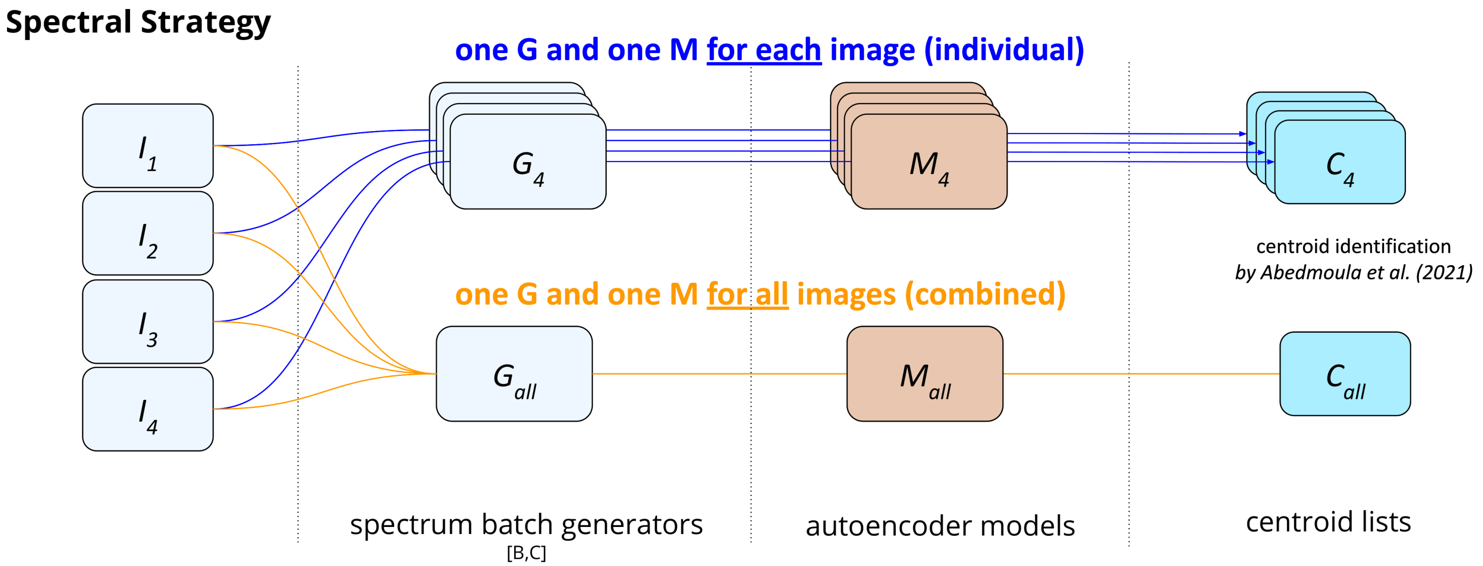

Fig. S3. Spectral Strategy: Example IV – pyM^{2}aia implementations of a spectral strategy for peak learning by Abdelmoula *et al.* (2021). Target objective is to learn how to reconstruct individual spectra of a set of MSI datasets I_n (four in the example) using an autoencoder model M. The result of the peak learning procedure is a list of centroids. Two different variants are illustrated. The upper blue path shows how to train four independent models M_n using four independent (individual) spectrum batch generators G_n of pyM^{2}aia. The lower orange path uses a single instance G_{all} of a pyM^{2}aia spectrum batch generator to process multiple images at the same time (combined).

Spectral Strategy: Example IV – autoencoder for peak learning All changes to the original peak learning code-base (Abdelmoula *et al.*, 2021) are available in a GitHub fork [3]. Results of the peak learning process are shown in Figure S6 and Figure S7. Different training strategies for individual and combined models are shown in Figure S3.

Spatial Strategy: Example V – ion-image-based co-localization All changes to the original self-supervised ion-image clustering code-base (Hu *et al.*, 2022) are available in a GitHub fork [4]. Results are illustrated in Figure S4.

Spatio-spectral strategy: Example VI – variational autoencoder In this example, the 3x3 spatial neighborhood of randomly selected spectra are used to train a variational autoencoder (see Figure S5). Results of encoded spectra (latent variable z) are shown in Figure S8.

Spatio-spectral strategy: Example VII – pixel-wise classification This example demonstrates the training of a pixel-wise classification model using spatial annotations on one MSI dataset, applying the trained model on unseen data of all four MSI datasets, and storage of the results including metadata for spatially correct display or further processing. Manual annotations were interactively created for a single sample (slice 3) and exported as a labeled image in NRRD format. Additionally, centroid lists were generated for each sample (slice 1-4) and combined into a single centroid list, exported in text format as comma-separated values (.csv). M^{2}aia was utilized for the interactive creation of labeled images and centroid lists. In the Python notebook of Example VII, we load all four imzML MSI datasets, including the labeled image (using SimpleITK) and the combined centroid list (using numpy). The list of centroids and the labeled image are then passed to the spectrum generator, generating batches of the form [X=[B,C,H,W], Y=[B]] (see Figure S5). Here, X represents the spectral data, and Y denotes the labels for each sample in the batch. We then build a convolutional neural network for classification using categorical cross entropy, a 9x9 spatial neighborhood, and randomly selected spectra from the provided annotated regions. Further details can be found in the example notebook.

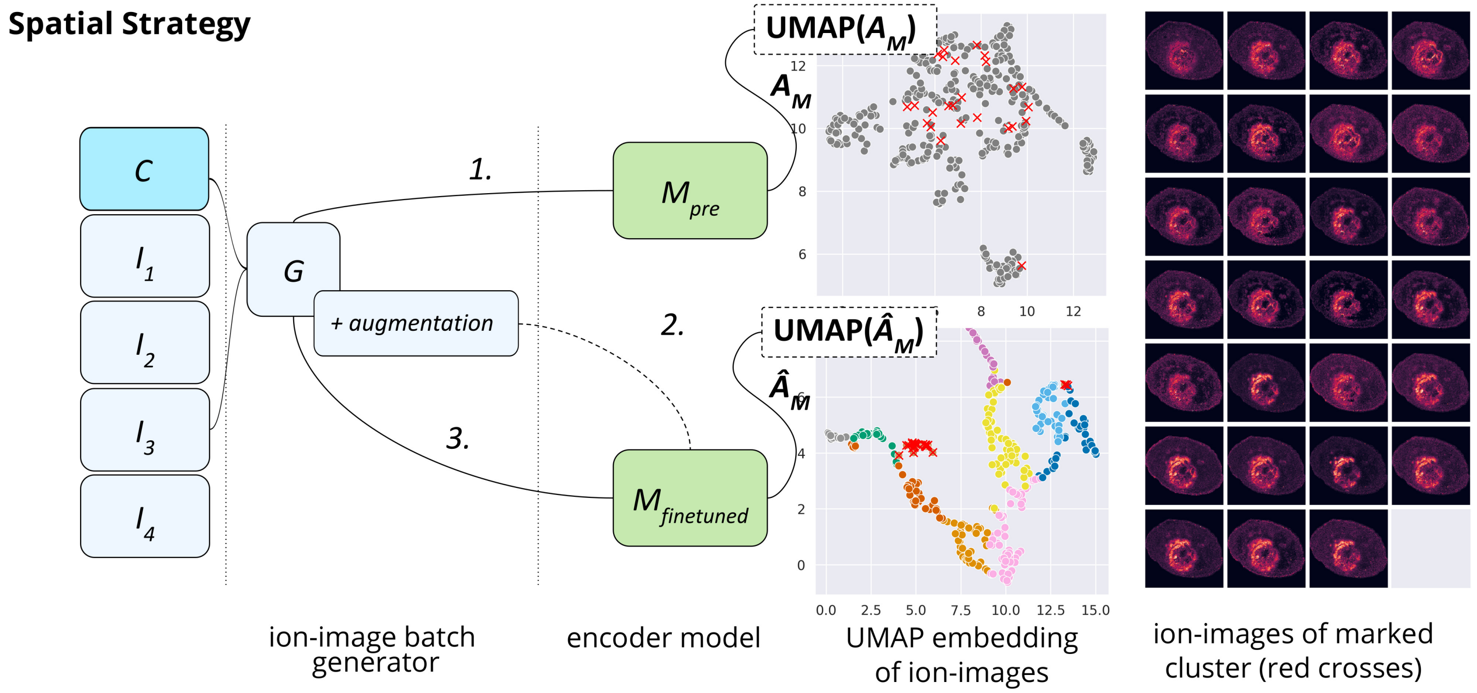

Fig. S4. Spatial strategy: Example V – pyM^{2}aia implementation of a spatial strategy for self-supervised clustering of ion-images by Hu *et al.* (2022). pyM^{2}aia's ion-image batch generator of MSI dataset I_3 is utilized to feed (1.) a pre-trained EfficientNet M_{pre} (Tan and Le, 2020) model to generate a lower (1024) dimensional embedding A_M of all ion-images generated with respect to a user defined list of centroids C. For (2.) fine-tuning of the model, unsupervised SimCLR (Chen *et al.*, 2020) training is applied, resulting in (3.) a refined embedding $\hat{A}_M$. Subsequently (fourth column in the figure), UMAP (McInnes *et al.*, 2020) is applied to embed A_M and $\hat{A}_M$ in two dimensions. Each embedded point refers to one ion-image. Spectral clustering (Damle *et al.*, 2019) was applied to $\hat{A}_M$. The resulting clusters are color-coded in the figure. To visually demonstrate that the clustering in the space $\hat{A}_M$ was successful, the ion-images corresponding to the cluster are marked with red crosses in the UMAP visualisations and are shown in the last column. As expected, the images are visually similar. Without fine-tuning, these images would not form a cluster, as can be seen by the wide distribution of the marked cluster instances in the UMAP(A_M) visualization.

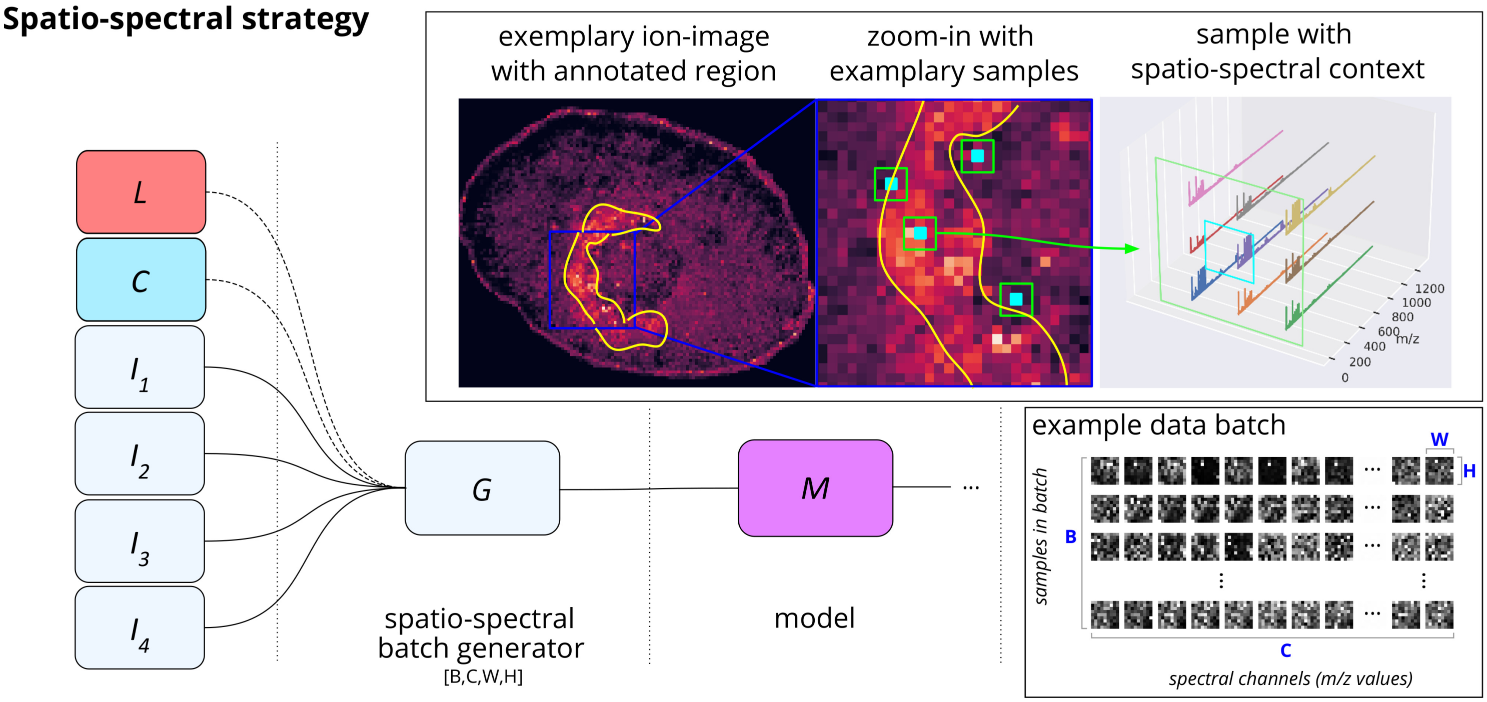

Fig. S5. Spatio-spectral strategy: Example VI/VII – variational autoencoder/pixel-wise classification. Generators can be initialized using label images (*L*) and/or centroid lists (*C*). For the description of the two examples, see the text. pyM^{2}aia enables individual as well as combined spatio-spectral processing of MSI datasets (as demonstrated for the spectral strategy in Example IV, see Fig. S3).

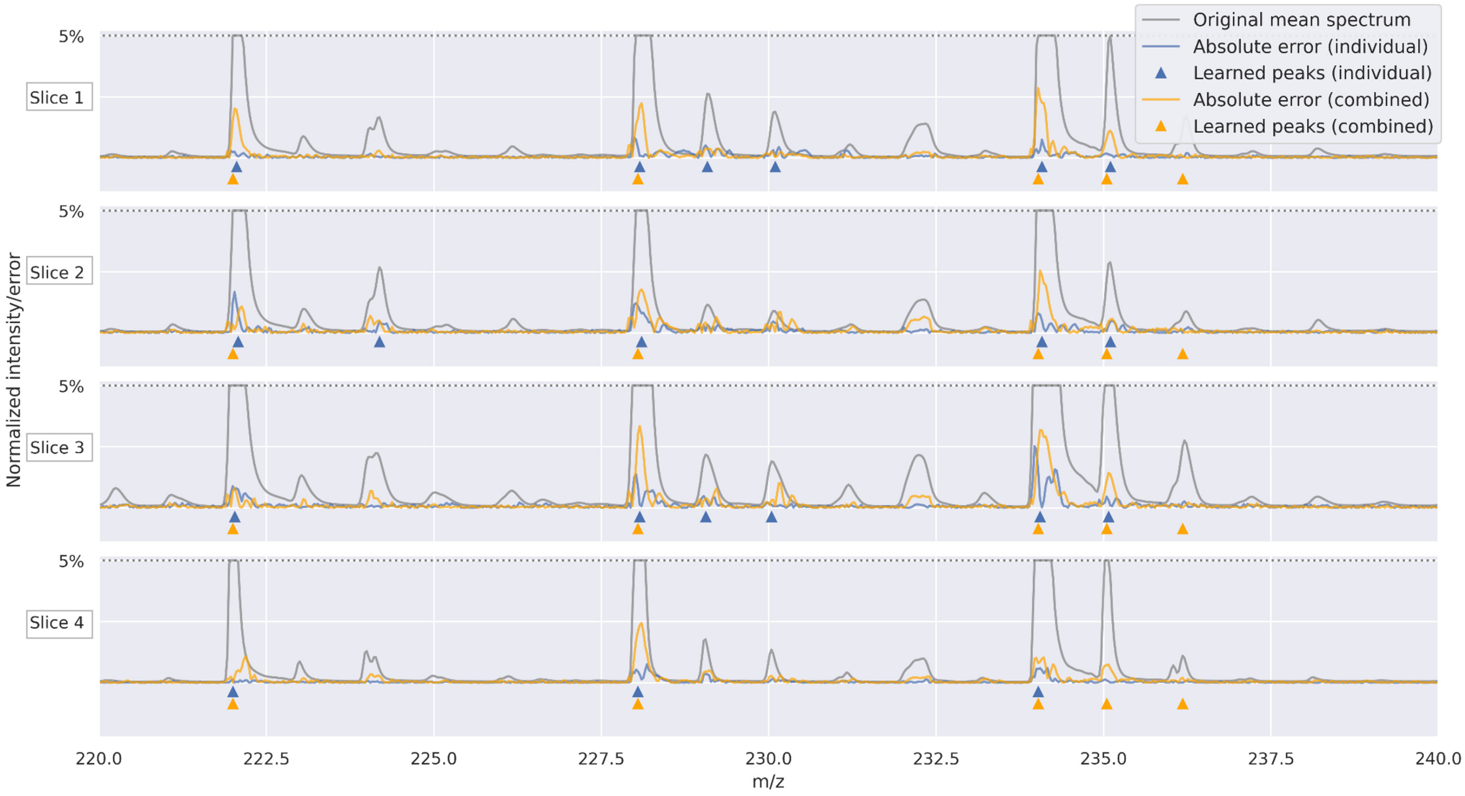

Fig. S6. Spectral strategy: Results of example IV – Peak Learning. Each row represents a single MSI dataset. Absolute errors between reconstructed mean profile spectra (individual models: blue lines; combined model: orange lines) and the original mean profile spectrum (gray lines) are shown for each slice. All values are normalized to 5% of the maximum of the respective original mean spectrum. Mass range *m/z* 220 - *m/z* 240. Learned peaks are shown for individual models (blue markers) and the combined model (orange markers)

.

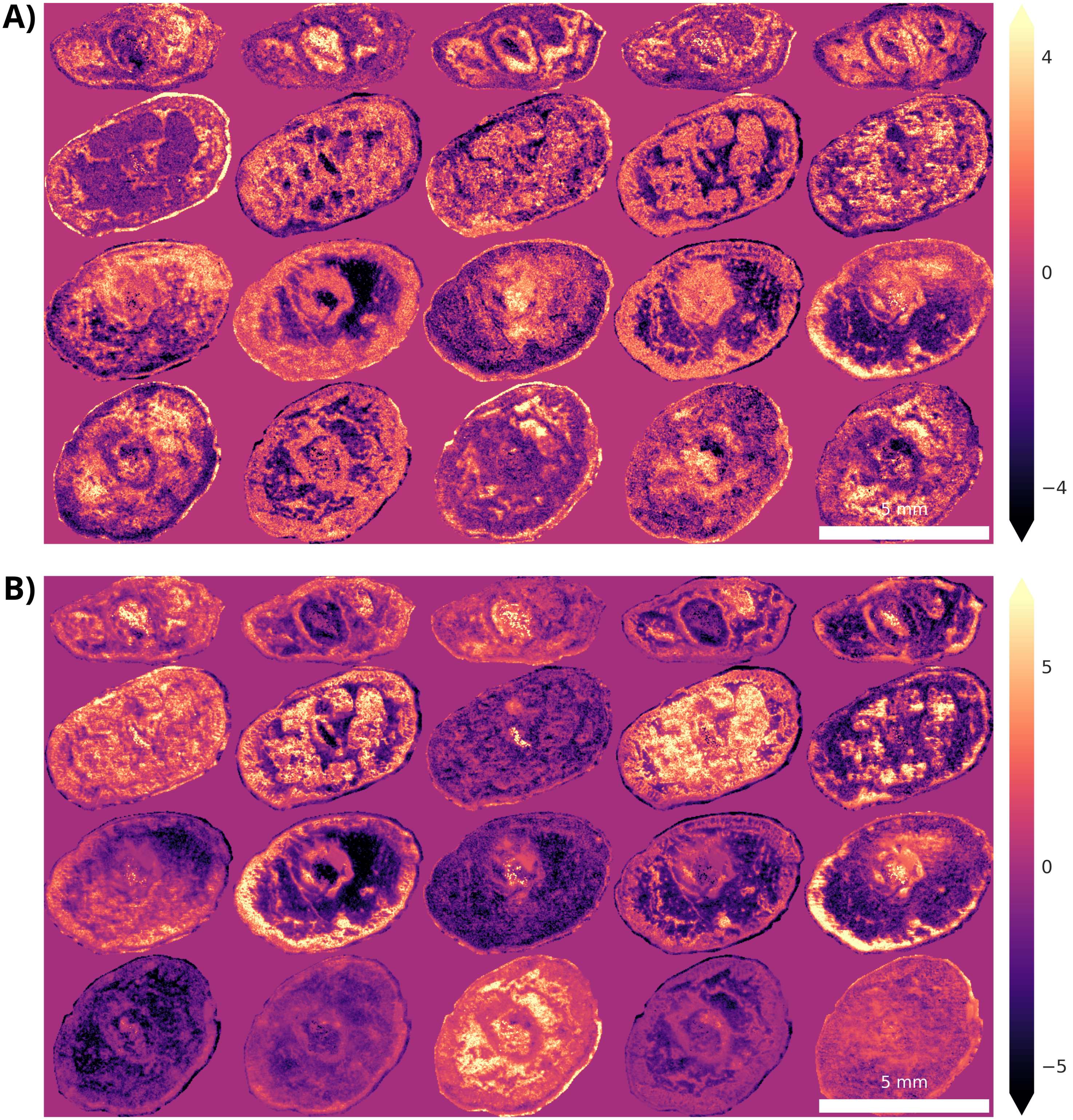

Fig. S7. Spectral strategy: Results of example IV – Peak Learning. Recovered structures of the original high-dimensional MSI dataset visualized with the represented values of the encoded spectra (latent variable z) of variational autoencoders. Each row represents a single slice. Each column represents one component of the encoded latent variable z. From left to right, each row represents the respective component of the latent variable z_0, z_1, z_2, z_3, z_4. In A) individual models and in B) the combined model is used to encode each spectrum of each MSI dataset. Displayed values represent data between the 1st and 99th percentiles.

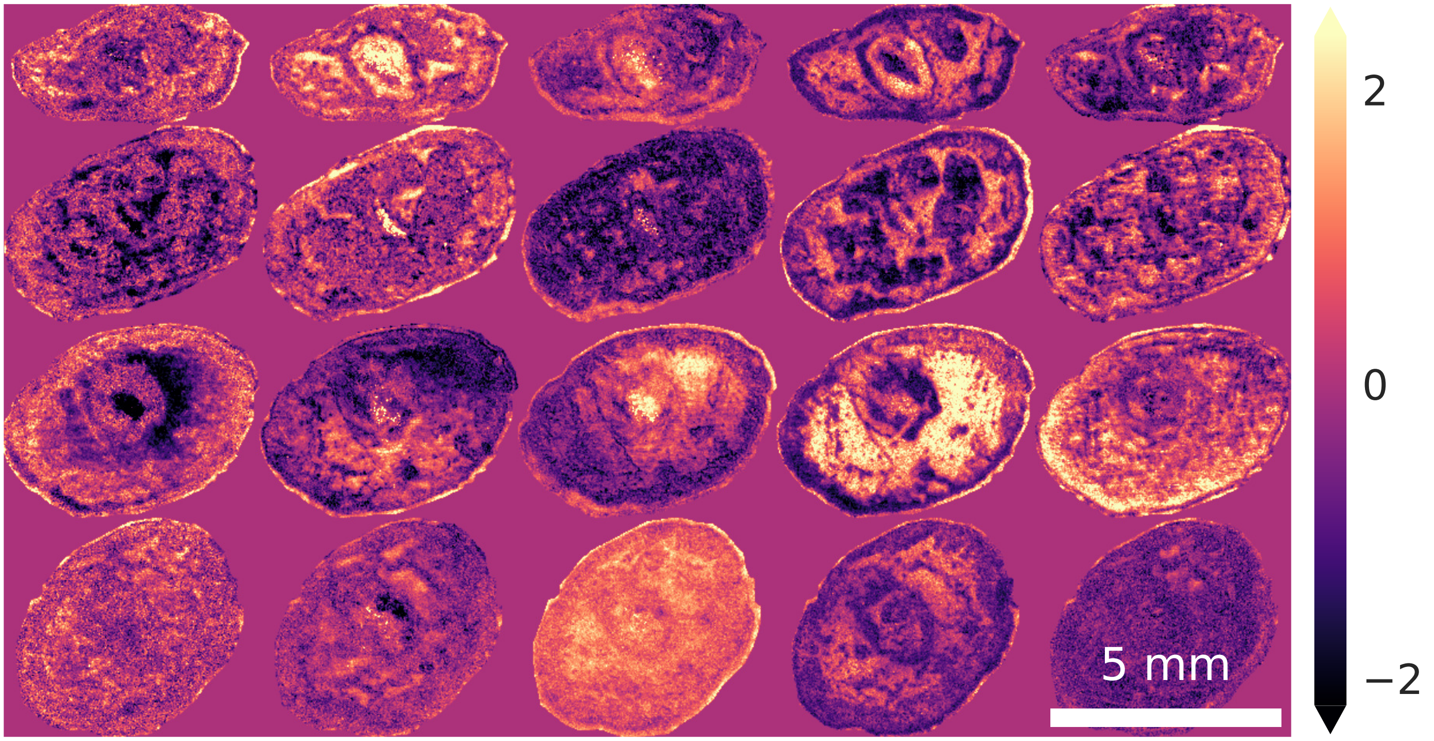

Fig. S8. Spatio-spectral strategy: Results of example VI – variational autoencoder. Latent variable z of the variational autoencoder using the spatio-spectral strategy. From left to right, each row represents the respective component of the latent variable z_0, z_1, z_2, z_3, z_4. Displayed values represent data between the 1st and 99th percentiles.

.

5. COMPARISON OF PYM^{2}AIA AND PYIMZML

Table S1 provides a comparison between pyM^{2}aia and pyimzML, as far as this is possible: pyimzML supports only loading of imzML datasets, whereas pyM^{2}aia additionally supports preprocessing (different methods for baseline correction, normalization, smoothing) and the data access functions for the three different strategies, which are therefore not directly comparable to pyimzML.

**Feature**	**pyM^{2}aia**	**pyimzML**
Lazy loading (low memory profile)	yes	yes
Spectrum access	yes	yes
Create ion-images	yes	yes
Signal processing	Normalization, Baseline correction, Smoothing, Intensity transformations	no (*)
Overview spectra	Mean, Max	no (*)
Normalization maps	TIC, Sum, Mean, Max, RMS, Internal	no (*)
Spectrum Generators (Spectral and spatio-spectral strategy)	continuous profile and centroid data	no (*)
Ion-image Generators	Spatial strategy, continuous/processed centroid and continuous profile data	no (*)
Average time to load all four datasets (**)	5.2 [seconds]	14.6 [seconds]
Average maximum memory usage (**)	640 [Megabyte]	577 [Megabyte]
ImzML meta-data queries	All XML elements with "IMS:.." and "MS:..." tags	Tags related to correctly represent the image (max count of pixels x/y, max dimension x/y, pixel size x/y/z)
Notes	* Not in scope of the package<br>** by default, M^{2}aia performs a full parse of imzML xml tags, creates an index image, normalization images (for all implemented normalization methods) and overview spectra (max/mean). For comparison with pyimzML, we implemented these functionalities directly in Python with numpy. All four data sets were loaded sequentially. The runtime and maximum memory usage was averaged over 50 repetitions.	

Table S1. Feature Comparison: pyM^{2}aia vs. pyimzML. System configuration: desktop PC, Ubuntu 22.04, AMD R © Ryzen 9 5900x CPU at 3.7 GHz 12-core processor, 32 GB physical memory, M.2 SSD, and Nvidia Titan RTX.

REFERENCES

Abdelmoula, W. M., Lopez, B. G.-C., Randall, E. C., Kapur, T., Sarkaria, J. N., White, F. M., Agar, J. N., Wells, W. M., and Agar, N. Y. R. (2021). Peak learning of mass spectrometry imaging data using artificial neural networks. *Nat. Commun.*, **12**, 5544.

Abdelmoula, W. M., Stopka, S. A., Randall, E. C., Regan, M., Agar, J. N., Sarkaria, J. N., Wells, W. M., Kapur, T., and Agar, N. Y. R. (2022). massNet: integrated processing and classification of spatially resolved mass spectrometry data using deep learning for rapid tumor delineation. *Bioinformatics*, **38**(7), 2015–2021.

Abu Sammour, D., Cairns, J. L., Boskamp, T., Marsching, C., Kessler, T., Ramallo Guevara, C., Panitz, V., Sadik, A., Cordes, J., Schmidt, S., Mohammed, S. A., Rittel, M. F., Friedrich, M., Platten, M., Wolf, I., von Deimling, A., Opitz, C. A., Wick, W., and Hopf, C. (2021). Spatial Probabilistic Mapping of Metabolite Ensembles in Mass Spectrometry Imaging. *bioRxiv*. doi.org/10.1101/2021.10.27.466114.

Behrmann, J., Etmann, C., Boskamp, T., Casadonte, R., Kriegsmann, J., and Maass, P. (2018). Deep Learning for Tumor Classification in Imaging Mass Spectrometry. *Bioinformatics*, **34**(7), 1215–1223.

Chen, T., Kornblith, S., Norouzi, M., and Hinton, G. (2020). A Simple Framework for Contrastive Learning of Visual Representations. *PLMR*, **119**, 628–635.

Cordes, J., Enzlein, T., Marsching, C., Hinze, M., Engelhardt, S., Hopf, C., and Wolf, I. (2021). M2aia—Interactive, fast, and memory-efficient analysis of 2D and 3D multi-modal mass spectrometry imaging data. *GigaScience*, **10**(7), giab049.

Damle, A., Minden, V., and Ying, L. (2019). Simple, direct and efficient multi-way spectral clustering. *Inf. Inference*, **8**, 181–203.

Geier, B., Oetjen, J., Ruthensteiner, B., Polikarpov, M., Gruber-Vodicka, H. R., and Liebeke, M. (2021). Connecting structure and function from organisms to molecules in small-animal symbioses through chemo-histo-tomography. *Proc. Natl. Acad. Sci.*, **118**(27), e2023773118.

Haug, K., Cochrane, K., Nainala, V. C., Williams, M., Chang, J., Jayaseelan, K. V., and O'Donovan, C. (2019). MetaboLights: a resource evolving in response to the needs of its scientific community. *Nucleic Acids Research*, **48**(D1), D440–D444.

Hu, H., Bindu, J. P., and Laskin, J. (2022). Self-supervised clustering of mass spectrometry imaging data using contrastive learning. *Chem. Sci.*, **13**, 90–98.

McInnes, L., Healy, J., and Melville, J. (2020). UMAP: Uniform Manifold Approximation and Projection for Dimension Reduction. *arXiv*. arXiv:1802.03426.

Ovchinnikova, K., Stuart, L., Rakhlin, A., Nikolenko, S., and Alexandrov, T. (2020). ColocML: machine learning quantifies co-localization between mass spectrometry images. *Bioinformatics*, **36**(10), 3215–3224.

Palmer, A., Phapale, P., Chernyavsky, I., Lavigne, R., Fay, D., Tarasov, A., Kovalev, V., Fuchser, J., Nikolenko, S., Pineau, C., Becker, M., and Alexandrov, T. (2017). FDR-controlled metabolite annotation for high-resolution imaging mass spectrometry. *Nature Methods*, **14**(1), 57–60.

Schramm, T., Hester, Z., Klinkert, I., Both, J.-P., Heeren, R. M., Brunelle, A., Laprévote, O., Desbenoit, N., Robbe, M.-F., Stoeckli, M., Spengler, B., and Römpp, A. (2012). imzML — A common data format for the flexible exchange and processing of mass spectrometry imaging data. *J. Proteomics*, **75**(16), 5106–5110.

Tan, M. and Le, Q. V. (2020). EfficientNet: Rethinking Model Scaling for Convolutional Neural Networks. *arXiv*. arXiv:1905.11946.

Thomas, S. A., Race, A. M., Steven, R. T., Gilmore, I. S., and Bunch, J. (2016). Dimensionality reduction of mass spectrometry imaging data using autoencoders. *IEEE Symp. Ser. Comput. Intell.*, pages 1–7.

Zhang, W., Claesen, M., Moerman, T., Groseclose, M. R., Waelkens, E., De Moor, B., and Verbeeck, N. (2021). Spatially aware clustering of ion images in mass spectrometry imaging data using deep learning. *Anal. Bioanal. Chem.*, **413**(10), 2803–2819.
